# Physicians’ Knowledge of Clinical Nutrition Discipline in Riyadh Saudi Arabia

**DOI:** 10.3390/healthcare9121721

**Published:** 2021-12-13

**Authors:** Khalid Aldubayan, Alhanouf S. Alsamani, Alanoud Aladel, Yara Almuhtadi

**Affiliations:** Department of Community Health Sciences, College of Applied Medical Sciences, King Saud University, Riyadh 11433, Saudi Arabia; alsamaanialhanouf@gmail.com (A.S.A.); aaladel@KSU.EDU.SA (A.A.); yalmuhtadi@ksu.edu.sa (Y.A.)

**Keywords:** physicians, dietitians, clinical nutrition, knowledge

## Abstract

Background: Nutrition plays a major role in the prevention and management of diet-related disease. With the absence of clinical nutrition dietitians, physicians are considered responsible for prescribing nutritional support. Identifying weaknesses in nutritional knowledge among Saudi physicians may provide guidance to improve their nutritional knowledge. Methods: A cross-sectional study that used an anonymous electronic questionnaire to investigate physicians’ knowledge of the clinical nutrition discipline. In addition to demographics, the questionnaire consisted of 15 questions covering six areas in the clinical nutrition discipline (macro- and micronutrients, nutrition and chronic diseases, nutrition and metabolic diseases, nutrition care process, nutrition support therapy, and research). For continuous variables, independent *t*-tests and one-way ANOVA were used. Results: A total of 332 had completed the questionnaire and were included in the study. Most of the physicians were Saudi (87%), male (73.5%), aged between 26 and 35 years (63.3%), and without health problems (56.3%). The mean score of the physicians’ knowledge was 5.3 ± 1.97 out of 15. Physicians who reported that they received some sort of nutritional training or course (M = 5.57, SD = 2.08) scored significantly more than physicians who did not (M = 5.10, SD = 1.86); *t*(330) = −2.174, *p* = 0.30. Conclusions: Nutrition should be reinforced as an important component of continuing medical education. There is a need for hiring more dietitians in health care settings in Saudi Arabia as an integral part of a multidisciplinary team delivering medical care services.

## 1. Introduction

The interest regarding effects of nutrition on health and disease has dramatically increased over the last decades. Nutrition is now believed to be a vital component of disease prevention and health promotion [1]. However, the prevalence of under/overnutrition and inadequate nutritional support is high among institutionalized patients in the Middle East [2] and Europe [3]. Malnutrition and low body mass index (BMI) were found to be the most common, underrecognized, and untreated conditions among hospitalized patients [3,4,5,6]. These may negatively affect every body organ, with consequences including reduced quality of life and increased morbidity and mortality rates [7,8]. For instance, malnutrition status correlates with an increased risk of death during hospitalization in central Europe in patients with heart failure [9,10].

Several systematic reviews, meta-analyses, and clinical trials have emphasized the important role of nutrition in the primary and secondary prevention of non-communicable diseases, which contributed to the development of nutritional guidelines and recommendations in various countries [11,12,13]. Moreover, delivering adequate nutritional support for hospitalized patients, particularly those in critical condition, has been shown to reduce the length of hospital stay, reduce health care costs, and decrease morbidity and mortality [14,15,16,17].

Malnutrition was estimated to range from 20% to 40% in hospitalized patients worldwide [3,4,18,19], and lack of nutritional knowledge among health care providers including physicians and nurses was one of the main factors behind this issue [20,21,22,23]. A Canadian study revealed that 42% of physicians who work in general practice consider their nutritional knowledge as weak [24]. Nutrition knowledge questionnaires of physicians working in general practice in San Diego, Canada, Qatar, and Kuwait revealed correct response rates ranging from 60% to 69% [24,25,26,27]. Interestingly, studies in Saudi Arabia showed that primary care physicians have scored the least, with an average of 51% correct answers [24,28].

Finally, medical doctors (MDs) are in several countries mainly responsible for prescribing nutritional support, and patients consider physicians to be one of the most credible sources of nutrition information [29,30]. Meanwhile, in some countries, clinical nutrition dietitians are responsible for nutrition care process [28]. Several studies have stressed the importance of providing and including nutritional courses within medical school curricula [21,31,32]. However, to date, the inclusion of clinical nutrition in the medical modules or training of specialized physicians, in addition to in the continuing medical education (CME) curricula intended for practicing clinicians, remains a low priority [15]. Identifying weaknesses in nutrition knowledge among Saudi physicians may provide guidance to improve their knowledge of nutrition in the future, in addition to encouraging medical schools to make nutritional courses mandatory instead of being optional. Therefore, this study aimed to assess physicians’ nutritional knowledge and to determine whether previous nutritional courses would make a difference in their knowledge in Riyadh, Saudi Arabia.

## 2. Materials and Methods

This was a cross-sectional study that used an anonymous electronic questionnaire to investigate physicians’ knowledge of the clinical nutrition discipline in Riyadh, Saudi Arabia. The questionnaire was distributed to the target population through pamphlet in health institutes and social media between February and March 2021. According to the Ministry of Health Statistical Yearbook 2020, there was 8601 physicians in the Riyadh region [33]. We assumed that half of them were in Riyadh city, as no clear statistic was found. A sample of 353 participants aimed to be reached based on a 95% confidence interval and 5% margin of error of the total population of physicians in Riyadh city. All physicians from different specialties, professional status, and gender who worked in Riyadh were included, while medical students and other health practitioners such as nurses, pharmacists, and allied medical sciences were excluded, in addition to physicians who worked outside of Riyadh.

The questionnaire consisted of 27 questions (12 characteristics and 15 quiz questions). The characteristics questions included physicians’ demographics such as age, gender, nationality, health condition, educational level, professional status, specialty, years of practice, and nutritional background. On the other hand, the quiz covered six areas in the clinical nutrition discipline, which were macro- and micronutrients, nutrition and chronic diseases, nutrition and metabolic diseases, the nutrition care process, nutrition support therapy (enteral feeding), and research in the nutrition field. Experienced researchers with clinical and academic backgrounds in the field of clinical nutrition formulated the 15 quiz questions to be tailored at the undergraduate level. The questionnaire was pretested for face validity, and the average time spent to finish it was 20 ± 2 min. The online questionnaire was set to be only one response per IP address, and it could not be continued once the participant left the questionnaire. In addition, the online questionnaire had the feature of showing a response timestamp and the total time taken to complete it. Therefore, any responses that took more than 25 min were excluded for validity reasons. The reason behind the time restriction was to increase the chance of receiving actual knowledge without going to references.

Data were statistically analyzed by using SPSS statistics 23 software (IBM Inc., Armonk, NY, USA). The participants characteristics were illustrated with counts and percentages. Descriptive statistics were applied for the clinical nutrition discipline knowledge score. Investigating differences in clinical nutrition knowledge among physicians’ characteristics was the primary study goal. Independent *t*-tests were used to measure any significant differences of two continuous variables, while one-way ANOVA was applied for more than two continuous variables.

## 3. Results

The online questionnaire reached 443 participants, and 403 participants completed it. A total of 40 participants were excluded due to exceeding 25 min in finishing the questionnaire (23 participants), working outside Riyadh (13 participants), and being in a health profession other than physician (5 participants). The study ended up with 332 eligible participants who completed the questionnaire. The demographics of the participants are illustrated in Table 1. Most of the physicians were Saudi (87%), male (73.5%), aged between 26 and 35 years (63.3%), and without health problems (56.3%). The profession of the majority was resident (45.2%) and specializing in internal medicine (27.7%). Approximately three-quarters of the physicians received their highest degree from an educational institute inside Saudi Arabia (77.4%). The majority of physicians were working in governmental hospitals (92.2%), were not working in academia (58.1%), did not receive any nutritional background (57.8%), and had working experience of less than 2 years (36.7%).

The counts and percentages of physicians who correctly answered each question of the fifteen questions that covered the six areas within the clinical nutrition discipline are illustrated in Table 2. Less than half of the physicians had correctly answered questions in the macro- and micronutrients area (43.3%) and in the nutrition and metabolic diseases area (42.8%). Around one-third of physicians had correctly answered the other four areas, which were nutrition care process (36.1%), nutrition support therapy (enteral feeding) (31.9%), research (30.1%), and nutrition and chronic diseases (29.8%). Moreover, the association between physicians’ demographics variables and their total knowledge score of the clinical nutrition discipline is shown in Table 3. There were no significant associations in all variables except the variable that showed whether the physician had previous nutritional training or a nutrition course. Physicians who reported that they received some sort of nutritional training or a course (M = 5.57, SD = 2.08) scored significantly more than physicians who did not (M = 5.10, SD = 1.86); *t*(330) = −2.174, *p* = 0.30. In addition, the mean of the 15 questions’ total scores was 5.30 ± 1.97.

## 4. Discussion

In current study, we selected a specific group of physicians of different specialties who we thought would be interested to a certain extent in clinical nutrition since they were working in departments where nutritional care is highly relevant. Of the 443 physicians reached, the majority responded, with a rate of 75% (*n* = 332). This rate was the highest among what was reported in several previously published studies [21,22,23,25,26]. Saudi male physicians comprised the highest proportion of the total population (87% and 73.5%, respectively). It was determined that 27.7% of participating physicians were specialized in internal medicine, and the others were distributed between surgery, pediatrics, ICU, obstetrics and gynecology, orthopedic, otolaryngology, emergency medicine, family medicine, medical interns, and other specialties. These differences however did not affect the response rate or the knowledge scores. Similarly, although the majority (36.7%) had less than 2 years of practice, independent *t*-tests did not show any statistically significant differences in either response rates or knowledge scores. This could indicate the importance of nutrition education programs or modules for inclusion in the medical curriculum for physicians [21]. Furthermore, of the 332 respondents, 92.2% were from governmental hospitals, whereas only 4.8% were from private hospitals, and 3% were divided between primary health care physicians and private clinics/centers. This also did not affect the results of the study.

The mean score for the correctly answered questions in the current study was considered fairly poor (35.3%) [27]. This is lower than what has been reported in the nutritional knowledge survey among Canadian physicians (63%) [24], Kuwaiti physicians (60%) [25], and residents from California (70%) [34]. However, our study’s questions were more about the clinical nutrition discipline than the general nutritional questions used by the other studies. In addition, the questionnaire in the California study consisted of only true–false questions, which give a 50% chance that would have raised the score far more than true values. On the other hand, other studies published by Al Numair [28], Al-Zahrani et al. [35], and Kirby et al. [34], which used multiple-choice questions, also demonstrated low scores ranging from 43% to 52% of correct answers. Lack of knowledge was found to be a major independent factor for insufficient nutritional practice; hence, there is a risk for malnutrition among hospitalized patients [20]. This may urge the need to improve nutritional knowledge among physicians working in departments involving nutritional care. In addition, this could also highlight the importance of integrating registered dietitians into the multidisciplinary team (MDT) for improving outcomes for patients with different medical conditions. Growing evidence have shown that dietetic care, delivered as part of the multidisciplinary approach, was significantly clinically effective and cost effective in the management of malnutrition and in improving hospitalized patients’ outcomes, such as decreasing mortality, speeding recovery, and shortening length of stay (LOS) [36,37,38,39,40,41,42,43].

Interestingly, the present study shows that physicians who previously had nutritional courses obtained significantly higher scores than those who did not (37.1% vs. 34% of correctly answered questions, respectively, *p* < 0.05). These results are in accordance with what Grammatikopoulou M. et al. observed, who found that having clinical nutrition specific courses was the only parameter responsible for increasing physicians’ nutritional knowledge rather than years of experience, working in academia, country of highest degree, gender, specialty, or professional status [20,21]. Unfortunately, physicians’ knowledge was previously assessed in Saudi Arabia, and studies show that this knowledge has not improved since 2004 [23,28,35]. This study is the largest among Saudi studies, yet shows similar figures. These results, however, stress the need for introducing mandatory nutrition education programs within the medical curriculum. The Council of Europe-Committee of Ministers has considered insufficient nutritional education to be a major barrier for adequate nutritional care [44]. Insufficient education could justify the inadequate nutritional knowledge observed in the current study. This lack of nutritional knowledge could suggest the low priority nutrition has, which is aligned with other studies from the USA and Europe [45]. Given the need for providing high-quality health care, knowledge of clinical nutrition should be encouraged in order to avoid omissions or critical mistakes that could be easily avoided. Several efforts and initiatives have been commenced to promote nutritional education worldwide, and many strategies have been proposed to overcome obstacles in nutritional training [46]. Nonetheless, the number of medical students rating their nutrition preparation as inadequate was found to be significant [47], even though several strategies have been recommended to overcome this issue [48].

A European Expert group published several recommendations to overcome the inadequate nutritional practice and knowledge in Europe [49]. One of these suggestions is to continue an educational program that covers general nutrition and nutritional support techniques for all healthcare workers. Furthermore, an improvement in self-efficacy might not be observed or show significant change with education by lecturing only, as it has limited opportunities for clinical and practical experiences [50]. Role modelling, role playing using either real or simulated patients, and discussing videos and web-based cases have resulted in better self-efficacy of the physicians, in addition to changing their attitudes toward nutritional care [51,52]. In addition to that, numerous studies have proved that the presence of a physician nutrition specialist (PNS) is a significant factor for effective nutrition training for physicians [53,54,55]. In a family practice residency program, a significant increase in the nutrition knowledge of resident physicians was observed when a nutrition education program was provided by a clinical nutrition specialist [34,56,57]. Given these findings, the American Society for Clinical Nutrition has recommended a full-time faculty member with expertise in nutrition to be employed in each main medical center. This can be used as a model for incorporating nutrition into patient care and advocating for changes in medical schools and residency programs [58]. More research is needed to determine the most effective ways to incorporate nutrition into the medical curriculum. According to Dalen and Alpert [59], nutrition classes in the pre-med curriculum are indisputably more relevant to future medical practice than organic chemistry, a variety of medical history, or foreign-language courses, which may be optional rather than mandatory. More instructional programs at all levels, the formation of nutrition support teams, and the separation of unique tasks between doctors, nurses, and dietitians could improve the delivery of efficient nutrition support and lower the risk of malnutrition in patients [60]. Registered dietitians are uniquely qualified to provide critical advice on illness prevention and health promotion [1,61,62]. However, because dietitians are not employed in all health-care settings in Saudi Arabia, all health-care workers should improve their clinical nutrition knowledge.

## 5. Conclusions

To conclude, the findings of the study show that primary care physicians require further nutrition education. Nutrition, on the other hand, should be a primary focus of continuing medical education. As the nutrition care process is essential for patient care, especially hospitalized critically ill patients, it is recommended to hire more clinical dietitians in medical settings in Saudi Arabia as part of a multidisciplinary team to ensure effective delivery of services.

## Figures and Tables

**Table 1:** Demographics (*n* = 332).

Variables		Count (%)
Age	≤25 Years	44 (13.3%)
	26–35 Years	210 (63.3%)
	36–45 Years	52 (15.7%)
	46–55 Years	18 (5.4%)
	56–60 Years	6 (1.8%)
	≥61 Years	2 (0.6%)
Nationality	Saudi	289 (87%)
	Non-Saudi	43 (13%)
Gender	Male	244 (73.5%)
	Female	88 (26.5%)
Country of Highest Degree	Inside Saudi Arabia	257 (77.4%)
	Outside Saudi Arabia ^1^	75 (22.6%)
Personal Health Problems Related to Nutrition	Without Health Problem	187 (56.3%)
	With Health Problem ^2^	145 (43.7%)
Specializations	Internal Medicine	92 (27.7%)
	Surgery	26 (7.8%)
	Pediatrics	22 (6.6%)
	Intensive Care	50 (15.1%)
	Obstetrics and Gynecology	9 (2.7%)
	Orthopedic	4 (1.2%)
	Otolaryngology	2 (0.6%)
	Emergency Medicine	9 (2.7)
	Family Medicine	35 (10.5%)
	Medical Intern	50 (15.1%)
	Others	33 (9.9%)
Professional Status	Medical Intern	50 (15.1%)
	Resident	150 (45.2%)
	Specialist/Registrar	34 (10.2%)
	Senior Registrar/Assistant Consultant/Associate Consultant	34 (10.2%)
	Consultant	64 (19.3%)
Type of Work Facility	Governmental Hospitals	306 (92.2%)
	Private Hospitals	16 (4.8%)
	Primary Health Care Centers	5 (1.5%)
	Private Clinics/Centers	5 (1.5%)
Working in Academia	Yes	139 (41.9%)
	No	193 (58.1%)
Years of Practice	≤2 Years	122 (36.7%)
	3–5 Years	97 (29.2%)
	6–10 Years	51 (15.4%)
	11–20 Years	45 (13.6%)
	21–30 Years	13 (3.9%)
	≥31 Years	4 (1.2%)
Received Nutritional Training	Yes	140 (42.2%)
	No	192 (57.8%)

^1^ Canada, Egypt, USA, UK, Netherlands, Others; ^2^ Overweight/Obesity, Cardiovascular Diseases, Diabetes Mellitus, Gastrointestinal Diseases, Others.

**Table 2:** Number and Percentages of Correctly Answered clinical nutrition discipline knowledge questions among Saudi Physicians (*n =* 332).

NO.	Area	Question	Correct Answer	Count (%)	Average (%)
1	Macro and Micronutrients	If you consume 2000 calories a day and want to limit the amount of fat you eat to no more than 30%, how many grams of fat is that?	67 g	111 (33.4)	144 (43.3)
2		Which of the following micronutrients is deficient among obese individuals?	Vitamin D	222 (66.9)	
3		Which is the predominant protein in human milk?	Whey protein	110 (33.1)	
4		Rana is diagnosed with celiac disease, and though would be expected to have deficiency due to fat malabsorption.	Cholecalciferol	132 (39.8)	
5	Nutrition and Chronic Diseases	What is the recommended protein intake for a diabetic patient on dialysis?	0.6–0.8 g/kg bodyweight	106 (31.9)	99 (29.8)
6		Which of the following statements is incorrect about Glycemic Index (GI)?	The GI ratings of food you eat, rather than the amount of carbohydrate you eat, has a greater influence on blood glucose levels after meals	92 (27.7)	
7	Nutrition and Metabolic Diseases	Sara is a 12-year-old patient with Von Gierke disease or Type I glycogen storage disease (GSD I). She has been always suffering from morning hypoglycemia. Thus, the best dietary recommendation would be:	A combination of eating cornstarch and complex carbs	142 (42.8)	142 (42.8)
8	Nutrition Care Process (NCP)	A 71-year-old female 6 months ago has endoscopically diagnosed reflux esophagitis, complaining of epigastric pain, vomiting, and decreased food intake, with 9 kg significant weight loss. The best clinical indicator to properly interpret the nutritional status in this case is	Subjective Global Assessment (SGA)	120 (36.1)	120 (36.1)
9	Nutrition Support Therapy (Enteral Nutrition)	You are determining the energy intake target for a 53-year-old, critically ill, male patient who is about to start enteral feeding. He is 170 cm in height and weighs 150 kg. His (BMI) is 51.9 and his ideal body weight is 70 kg. Body temperature is 37.3 degrees Celsius and minute ventilation is 12.5 L/min. What goal energy value would you use as the basis for the feeding plan?	25 kcal per kg ideal body weight	89 (26.8)	106 (31.9)
10		The most appropriate enteral formula to start with for a patient with short bowel syndrome is?	An isotonic, standard, fiber-containing formula administered via continuous gastric infusion	87 (26.2)	
11		A patient was admitted to the hospital with advanced lung cancer. He had been unable to take any food orally (NPO) for two days and was supported with IV fluids. His weight at admission was 53 kg and his height 172 cm. What protein value would you use as the basis for the enteral feeding plan?	1.2–2 gm per kg	106 (31.9)	
12		The best scenario to develop in maintaining enteral feeding intolerance, when gastric residual volumes (GRVs) 250–500 mL.	Assess other signs of intolerance > if observed hold feeding for one hour and recheck > Promotility agents > Restart at highest tolerated rate > Glycemic control evaluation > Reassess enteral feeding	142 (42.8)	
13	Research	In a randomized controlled trial for investigating the possible effect of a nutritional supplement on a disease, allocation concealment can minimize.	Selection bias	137 (41.3)	100 (30.1)
14		What is the appropriate validity technique to validate the content of a food frequency questionnaire?	Criterion	109 (32.8)	
15		From the following statements, one is true about analytic nutritional epidemiological studies.	The researcher has control over the variables	54 (16.3)	

**Table 3:** Association between the mean of total nutrition score and the physicians’ demographic variables.

Variable	Clinical Nutrition Discipline Knowledge Mean Score (SD ^1^)	*p*–Value
Total (*n* = 332)	5.30 (1.97)	
**Received Nutritional Training ^2^**		
Yes (*n* = 192)	5.57 (2.08)	0.030
No (*n* = 140)	5.10 (1.86)	
**Years of practice ^2^**		
≤2 Years (*n* = 122)	5.35 (2.05)	
>2 Years (*n* = 210)	5.27 (1.92)	0.702
**Working in Academia ^2^**		
Yes (*n* = 139)	5.37 (1.85)	
No (*n* = 193)	5.25 (2.05)	0.590
**Highest Degree ^2^**		
Inside Saudi Arabia (*n* = 257)	5.26 (1.97)	
Outside Saudi Arabia (*n* = 75)	5.44 (1.95)	0.479
**Gender ^2^**		
Male (*n* = 244)	5.37 (1.96)	
Female (*n* = 88)	5.09 (1.99)	0.249
**Physicians Specialties ^3^**		
Intensive Care (*n* = 50)	5.68 (2.12)	
Family Medicine (*n* = 35)	5.34 (1.89)	
Pediatrician (*n* = 22)	5.32 (1.91)	0.661
Internal Medicine (*n* = 92)	5.20 (1.96)	
Others (*n* = 133)	5.21 (1.97)	
**Physicians Professional Status ^3^**		
Consultant/ Associate Consultant/Assistant Consultant/ Senior Registrar/Specialist/ Registrar (*n* = 132)	5.45 (1.85)	0.408
Resident (*n* = 150)	5.25 (2.01)	
Medical Intern (*n* = 50)	5.04 (2.12)	

^1^ Standard deviation; ^2^ Associations between variables and nutrition mean scores were tested by the independent *t*-test with equal variances according to Levene’s test; ^3^ Associations between variables and nutrition mean scores were tested by one-way ANOVA with homogeneity of variances according to Levene’s test.
